# Porcelain Art in Dentistry

**Published:** 1904-07

**Authors:** Herbert L. Wheeler

**Affiliations:** New York, N. Y.


### PORCELAIN ART IN DENTISTRY.

By Herbert L. Wheeler, D. D. S., New York, N. Y.

(Read before the New Haven Dental Association, at its first annual convention, New Haven, Conn., March 15, 1904.)

Porcelain art in the realm of operative dentistry probably began with the operation of Dr. A. J. Volck of Baltimore, which consisted of grinding a piece of already baked porcelain to approximately fit the cavity, and setting it as an inlay by packing gold around between the porcelain and the cavity margins. This method is first described by him in the *American Journal of Dental Science* for July, 1857, wherein he credits the suggestion to Professor Maynard of Washington.

This plan has been employed, in cases where the operator has had the skill and patience to grind a piece of porcelain to an accurate fit, down to the present time, though oxyphosphate cement has been substituted for the gold in fastening the inlay.

I have seen, within two years, a beautiful specimen of this work, in which a broken tip of a central incisor had been restored by Dr. J. Morgan Howe, twenty or more years ago, and the porcelain and its fastenings were still in perfect condition. This tip was made from an Ash & Sons tooth. One advantage in using porcelain already baked is that in all probability it has been more scientifically handled and annealed by the expert workman employed by the manufacturer than modern inlays are by the average dentist; hence it is likely to be of superior texture and strength, and, if accurately fitted, will undoubtedly do as good or better service than many of the inlays baked by inexperienced men in the profession today. Its great drawbacks are difficulty of getting a perfect fit and the length of time required for the operation.

The present method of making porcelain inlays to fit the cavity by means of fusing porcelain, glass, etc., in platinum or gold matrices which have been previously made to fit the cavity by pressure and burnishing, was originated by Dr. Wm. Rollins of Boston, and first described by him in a paper read before the Society of Oral Science in June, 1880. Dr. Rollins was also the first to use gas furnaces for baking porcelain.

### INGREDIENTS OF PORCELAIN BODIES.

Chemically speaking, the high and low-fusing materials are the same, the difference between them being one of proportions in the ingredients rather than of dissimilarity in the materials, except that all of the low-fusing bodies contain artificial flux, which greatly increases the soluble elements in the body. The probabilities are that the Jenkins low-fusing body has been changed several times, but despite this fact it is, I think, a glass, though it has been claimed that it is made by the ingenious mixture of several feldspars. That it is composed,—if this be so,—of different feldspars whose chemical formulæ may vary slightly because of different proportions of potassium or sodium, does not change the fact that the entire body is fusible, save perhaps a very small proportion of kaolin, which, so far as I can see, does not add to the strength or quality of the material, and greatly increases its shrinking capacity. It is not impossible that a body made in this way, though it be low-fusing and a glass rather than a true porcelain, may, if properly annealed in the cooling process, make a very strong substance. Its weakest point lies in the possibility of its being eventually affected by the fluids of the mouth, owing to the large amount of soluble salts, such as potassium, sodium, or magnesium, used to lower the fuing-point.

The high-fusing materials differ from this in that they contain a greater or less proportion of silica, which is infusible under ordinary circumstances, but may become fusible at a high heat, through grinding too fine or through adding too large a proportion of the highly alkaline constituents.

All of the high-fusing bodies, so far as I have observed, are true porcelains, containing a sufficient quantity of silica, along with the feldspar and kaolin in their make-up, to give stability and strength to the material.

The greater the proportion of silica, so long as there is enough feldspar to bind the particles closely together, the higher the fusing-point, and if proper care be used in cooling, the greater the strength of the material. Both for the purpose of producing proper form in lost tooth-structure and for obtaining strength, I consider the high-fusing material much superior to the low-fusing.

I do not agree with some writers who favor high-fusing material, that the better breaking up of the rays of light by the high-fusing body in all cases produces a more natural-looking material.

The higher-fusing the body used, the more stable the colors and the less danger of bubbling. These advantages are due to the lessened amount of alkaline material contained in the high-fusing bodies.

### STRENGTH OF INLAY PORCELAINS.

From some practical experiments which I have made, with the expenditure of much time and patience, I am inclined to the belief that the strength of the high-fusing as well as that of the low-fusing material is somewhat affected by the quality of the feldspar used in the flux, and it has occurred to me that possibly the chemical changes produced when the heat is carried to a point where the color disappears may also affect the strength of the material. A feldspar is a silicate of alumina, with silicates of other bases, either soda, potash, or calcium. These soluble alkaline portions vary considerably in different feldspathic rocks.

I have come to these conclusions from observing the tensile strength of the different inlay materials upon the market. In order to obtain the following results I made blocks of the different inlay materials about one-half inch long, three-eighths inch wide, and three-sixteenths inch thick, putting these in a mcahine for testing the strength of teeth.

The Jenkins material will stand an average strain of 32½ lbs., while it will go a little higher—to 37½ lbs.—if the material be baked so that it rounds almost to a globule and the color is lost to some extent. Ash & Sons' low-fusing stood a strain of 30½ lbs., and their high-fusing 45 lbs., this high average being brought about by one remarkable block which stood a strain of 60½ lbs. The Consolidated inlay material, which was originally made by Mr. Whiteley, now with the Dentists' Supply Co., averages 39 lbs., while the body now made by Mr. Whiteley averages 41 lbs., and the Brewster high-fusing enamel, in which the silica is apparently ground to extreme fineness, averages 19 lbs. The S. S. White high-fusing porcelain averages 33½ lbs. Parker's body, which is something like the following formula—Spar 4 oz, silex 3 oz, kaolin 1 dwt.—would not break, the platinum pins breaking instead. These tests, which were very carefully made by myself at different times, seem to indicate as a whole that the high-fusing, both for strength and for the obtaining of satisfactory colorings, are vastly superior to the low-fusing bodies.

### FUSING-POINTS OF PORCELAINS.

Upon going over the fusing-points given us by Mr. Hammond, I decided to make some tests with the pyrometer for myself. In these I used colored glasses in order to observe the operation of baking more closely and accurately. This plan protects the eyes from the glare of the furnace.

I obtained the following results, some of which vary from those given by Mr. Hammond. The degrees are given in the Fahrenheit scale:

| | | |
|---|---|---|
| Jenkins' enamel, | 1552° | Fahr. |
| Ash & Sons' low-fusing, | 1580° | " |
| Ash & Sons' high-fusing, | 2084° | " |
| Consolidated, | 2084° | " |
| Whiteley's, | 2228° | " |
| Brewster's, | 2084° | " |
| The S. S. White Co.'s, | 2228° | " |
| Parker's, | 2588° | " |

A body which Mr. Whiteley sent for me to try fused at 2264°, and held its color the best of anything in the list except Parker's. This also stood, in the tensile strength tests, an average strain of 48 lbs., and one piece refused to break, the pins breaking first.

In the matter of shrinkage, the Parker body contracted the least and showed the least tendency to warp. The shrinkage of the S. S. White and the Whiteley bodies was nearly the same, the former shrinking a little the more. This may be because of starch or gum tragacanth being put in the S. S. White body to make it work easier.

The Consolidated, the Ash & Sons' high-fusing, and Brewster's were about the same, the Consolidated shrinking a little less than the other two. Next came Ash & Sons' low-fusing, and the greatest shrinker of all was Jenkins' enamel; this also showed the greatest tendency to change shape by warping or drawing up at the ends. It would seem that the time required to work the Jenkins' enamel successfully must be greater than any of the other enamels or porcelains, though there is doubtless some time gained by the furnace not being so highly heated to cause fusing.

The Ash & Sons' low-fusing body seems to be much nearer a true porcelain than the Jenkins' enamel. Their high-fusing body and that made by Brewster have some similar characteristics. I apprehend that the infusible silica in both these bodies is very finely ground, though the Ash material seems to be able to withstand twice the strain that Brewster's does.

These results seem to indicate that for most cases of inlay work all the materials on the market may be of sufficient strength, and that with some slight modifications the higher the fusing-point of a body the more certainty of its color withstanding the heat of baking, and the greater possibilities of artistic work in those difficult cases where it is necessary to produce a concavo-convex surface in order to carry out a resemblance to the natural teeth.

Generally speaking, the higher the fusing-point the greater the strength, but this is materially affected by the care with which the heated porcelain is protected from changes of temperature, such as currents of air, during the cooling process, and by the rapidity with which this process is carried on.

No porcelain or glass material can be taken from a heated furnace without greatly lessening its toughness and edge strength. Still there remains this undisputed fact: The lower you get your fusing-point, the nearer you come to a glass compound rather than porcelain, and the greater the probability of the surface of the enamel being unfavorably affected by the fluids of the mouth.

Thus far the Jenkins' enamel is the only glass-like material that is still much in vogue, and from cases which I have seen in which the color has changed and the surface become roughened after having been awhile in the mouth, I believe that due caution is not being exercised by our profession in adopting a low-fusing material before it has been demonstrated by the test of time that it is absolutely insoluble in the fluids of the mouth. It seems to me that there is no good reason why the inlay material furnished by the manufacturers should not be fully as strong and keep its color as well as the artificial teeth they produce.

### AN AID IN SECURING DENSITY.

It was suggested by Dr. Littig of New York, in discussing a paper read before the New York Institute of Stomatology by Dr. Robert Moffatt last October, and published in the January *International Dental Journal,* that manufactured teeth made in molds were stronger than carved teeth because of pressure used in bringing the molds together.

By experiment I found that by a gentle tapping of the instrument which held the matrix on which was placed the porcelain body by another instrument, and a careful absorption of the moisture by blotting paper as it comes to the surface, until no more moisture can be extracted, a much greater density is secured, and consequently less shrinkage and greater strength than when the minute particles are forced into place by pressure; thus demonstrating that, given the same material as is used for artificial teeth, a greater density and strength can be secured, the conditions of baking being the same, by bringing the particles of the porcelain body into the closest relation with each other by gentle tapping rather than pressure.

I would suggest that if you wish to do an sepecially strong piece of work that may be more lasting and of better shade, this might be done by securing a tooth of the required color, pulverizing it in a mortar, and baking it as an inlay. This will avoid dependence upon any particular make of body, and will doubtless prove as effective a way of obtaining the desired shade as the attempted blending of the various colors sold in an inlay set.

*Discussion.*

Dr. W. A. Capon, Philadelphia—Tensile strength I will touch on for the simple reason that the greatest test of any porcelain is in actual use in the mouth. These tests made out of the mouth pulling the pins out of teeth etc., do not amount to much, as it is applying the force in a manner that is never experienced in the mouth. It is a pulling force, while in the mouth the strain is in a pushing, grinding direction. If someone would invent a device by which the force could be applied as in the mouth, these tests might amount to something. I care not whether the tensile strength of porcelain be twenty-five, thirty, or thirty-eight pounds, if the lowest will stand the strain in the mouth.

With regard to the fusing-point of porcelain, it is not the fact of its fusing at so very high or low a temperature that gives it the necessary strength. Touching that point, I think this was ascertained by Mr. Hammond and myself in my own laboratory, and the results are not astray to any great extent—they may be two or three degrees off, but the man has to be a very accurate workman that will take a material with a fusing-point of 2400° and get exactly the same result as somebody else. For instance, I make the fusing-point 2400° in my furnace, and he makes it 2380° in his; that practically makes no difference in the temperature. When you get nearer the fusing-point of gold it does make a difference, however, as that difference in the fusing-point would destroy your matrix. In the low-fusing bodies, if you allow the heat to go twenty points beyond the fusing-point, you are likely to affect the coloring matter, and that is the latitude of the low-fusing bodies. In the high-fusing it would take from forty to fifty degrees greater heat before affecting the coloring matter, therefore you have a greater latitude of variance in the high than in the low-fusing bodies. If you are using the low-fusing bodies, the heat has to be trained just exactly right, or you will get a change in the color. But the loss of shade is very easily overcome by experience.

I take exception to the essayist saying that the texture of porcelain is of equal degree of density when tapped as when subjected to pressure, and for proof of that you can take any material you may wish to, and do as he says, and take the same material and subject it to pressure; then brek the two materials, and you will find that the texture of these materials is different, and that it cannot be possible for the particles to lie as closely together as when forced together by pressure. Another proof is to take an ordinary pressed tooth and grind the surface and it will show a very close texture, practically smooth. With four-fifths of the porcelain bodies, after baking and grinding in this way, you do not have an even surface. This is a point that is practical, and one reason that I speak of it is because that in grinding the porcelain after it is in the mouth you have not the same opportunity of getting a smooth surface as in the pressed tooth.

With regard to the extremely high fusing-points I do not think there is any necessity of going to extreme temperatures to get the stronger material, and I think anywhere in the neighborhood of 2200° or 2300° is sufficient for our use. For an illustration of that, several years ago we had nothing but the Close's body to deal with. A few weeks ago I had occasion to renew a piece of bridge work made by myself a number of years ago. It was a jacket crown for one abutment and a porcelain-covered platinum crown for the other. I made the extension by running platinum wires from the jacket crown to the other abutment, building porcelain on this, and finally the whole mass was fused together. After years of constant use it had dipped a little between the abutments. I looked up my books, and found that this piece of work had been in for eleven years. I worked for fifteen minutes to get that jacket crown off, and the porcelain came off piecemeal; the last thing to come off was the veneer, but the porcelain in that was just as strong as it could be. That was Close's continuous-gum body, and was fued at 2300°. I do not want anything higher than that; it is not required.

With regard to the question of shadows, I think the result in that line is according to skill. I care not whether it is an old practitioner or a young man just entering the work, do not expect you are going to get the best results at once. If you are skilful, and have a tendency toward the artistic, porcelain work will come easy to you. There are plenty of dentists who can never become porcelain workers, simply because they are not adapted to the work. It is just the same with this work as it would be if we all were to try to reproduce a sketch now. There would be a few who could probably do it readily, but the great majority could not do it, yet most could acquire the skill. Just the same with this work; some can acquire the skill, and some cannot. As a proof of how difficult it is for some men to match colors, you see men go into a dental depot and when a tray of teeth is set before them they are thoroughly confused, and have to ask the clerk to select the proper shades for them. With regard to opacity, that is a bugbear to a great extent, because opacity is not in reality what we are after.

